# Perineal Injuries and Methods of Repair1Read at the 18th annual meeting of the American Association of Obstetricians and Gynecologists at New York, September 19-21, 1905.

**Published:** 1905-10

**Authors:** Joseph Price

**Affiliations:** Philadelphia, Pa., 214 N. Eighteenth St.


### Perineal Injuries and Methods of Repair.[1]

By JOSEPH PRICE, M. D., Philadelphia, Pa.

IN THE great city of New York a quarter of a century ago I received at the hands of the staff of the Woman's Hospital of this state the instruction I desired in plastic surgery. I shall never cease to express my appreciation of the kindness and attention shown me by Emmet, Thomas, Lee, Hunter, and Boseman, and their assistants. They were all greatly interested in plastic surgery,—perineal, cervical and the repair of fistulæ. They were

1. Read at the 18th annual meeting of the American Association of Obstetricians and Gynecologists at New York, September 19-21, 1905.

doing abdominal surgery as well, but they all did fine plastic work of which I desire at this time especially to speak. They all appeared to look to Emmet for the advanced or modified procedures for the repair of cervical injuries and all the accidents incident to parturition.

The gynecologists throughout the world were practising the old methods of perineal repair of Baker Brown, Simon and many others; Emmet was revising their procedures, studying and repairing the injuries of the pelvic floor or diaphragm, going deeper than the simple injuries of the external soft parts in both the study of the lesion and its repair. Large numbers of active practitioners and surgeons at home and abroad were visiting New York to witness the work of this great past master of plastic surgery. I have had an opportunity of seeing a great number of operations at home and abroad, but none compared with those of Emmet in completeness of method and result. I had tried about all of the old procedures,—the Baker Brown external operation I had done many times,—and I might just as well have told my patient to put on a pair of tightly fitting drawers. The Simon, Hildebrand, Freund and Fritz operations were no better.

The trefoil of Emmet or the Hegar were improvements over all the external procedures. At present the interest in plastic surgery is not quite so general as then, while the Woman's Hospital of New York, grand old school of plastic surgery lived. Our interest at present in the surgery of the right groin is too great for operators to spare time for this important study of plastic work. Recently I have recognised a welcome reaction. Only lately have I received communications of this nature asking my opinion of the value of deeply placed sutures before labor to prevent injury to the pelvic floor, levator ani and fascia, and one regarding the value of deeply placed post-partum sutures to restore the overstretched or retracted muscles and retrovaginal fascia. Again I find renewed interest throughout the country in the Emmet methods. In discussion of the subject I find that a large number of men have a clearer understanding of Emmet's literature; that they prefer and practise his inside operation for the restoration of the pelvic floor without modification, although some of the earlier pupils were inclined to modify and to tack "I," "me," and "my" to their procedures. The one,- two- and three-stitch methods are fortunately all dead. The flap splitting operation, an external procedure, is also largely abandoned.

I discuss the subject again here today to urge the importance of an early and more general practice of good surgery. All injuries or mutilations incident to parturition should be repaired early, while those in the perineum should be done primarily by the introduction of deep inside sutures. At the termination of the child-bearing period early or late relaxation of the pelvic floor should be corrected. Both deep external and internal injuries should be restored early to conditions that existed before childbirth. We could prevent physical and mental disturbances, displacements and procedentia, cysts and rectovaginocele by early repair; we should not wait for all the sequelæ and symptoms of injuries so easily recognised and repaired. Early posterior displacements following deep injuries of the levator ani and pelvic fascia prevent conception and favor abortions, while the constant pressure on the sensitive sacral plexus of nerves with the distressing backache and the sensation of something coming down and protruding starts a large number of women to rest cures and to the neurologist.

Fortunately the clinician or practitioner at present recognises the importance of such lesions and examines his patient or has her examined by someone interested in plastic surgery. Some of the most pleasing results I have ever had in gynecologic surgery have been in young women twenty-two to twenty-six years of age, with one or two children with some acute nervous disturbance following a deep injury and displacement. Some of them were about to be placed in asylums. Careful repair of the injuries in the birth passage restored them fully to their homes, their families and to active and useful lives. I rejoice that a good number of our abdominal specialists are paying more attention to the soft parts and to the deep pelvis and its contents.

The general surgeon has recently turned his attention from the appendix to the prostate. I am glad he is traveling down, not that his natural tendency has not been in that direction; but the schooling that he will get in the enucleation of that organ is just what he needs for deep pelvic surgery and its natural extirpations. Well done inside plastic surgery anticipates and relieves a good number of distressing symptoms, decensus, backache weight and pressure, vaginal respiration with its accompanied admission of dust and dirt admixtures, or normal menses and the decomposition that follows and the heat, burning and itching that results. I have known men to resect all the genital nerves they could find with their knowledge of anatomy, when in all probability good early plastic surgery would have prevented the symptoms for which they were operating and failing to relieve.

Early conceptions are very pleasing after plastic surgery. I make the discussion and draw my conclusions not so much from what I have accomplished, but from the good results and records of many of my friends. Patients and doctors fear recurrence of injuries, but when the results are fully stated I find about all are willing to have the repairs made many times and feel abundantly rewarded. I often tell them that a fine child will probably follow the repair and that a few sutures will not hurt them; that restoring them to anatomical ciditions that existed before childbirth influences them to submit to my counsel.

We have for a long time neglected plastic surgery; we are not giving the subject sufficient study; many of our methods and materials are defective. The appliances of the operating room of the Woman's Hospital of the State of New York were thrice better than those of the modern operating room. At home in my private hospital I have practised as closely as possible just what I was taught by Emmet and his pupils; using the low operating table; large low windows and the same instruments and materials. I found in traveling about the country and operating in the new costly operating rooms and on the rapid transit tables, that something was wrong and it worried me. I soon recognised that the table and windows were too high; that at home I looked down upon my work and away from home I had to look up to it in a bad light. Now, in view of my experience on this point, let me urge you to use a table and a light that will afford perfect command of the field of operation.

More prolonged preparations should be made for all plastic surgery; especially should suture of a torn cervix always precede perineal repairs. Free puncturing, twice or thrice, should be practised to relieve all cervical cysts and congestive hypertrophies, and add to this free douching and dressing the uterus forward for a few days, together with free purgation.

One operation or one object lesson by Emmet was not sufficient for the visitor. Large numbers of doctors left New York with a very cloudy mental picture of the procedure, and about all the attempts they made were unquestionable modifications of his work and what they saw. Some criticised the descriptions of his operations, asserting that they were not clear; that they could not understand them. If they got the stitches inside they rolled out. The denudation and suturing of the lateral sulci were not understood; they were all accustomed to working on the surface, that is, to doing skin surgery; like most surgery at that time it was difficult to tempt them to do deep work.

Had Emmet taught his refined plastic surgery a quarter of a century later, when surgeons had given up dermatology and invaded all the principal cavities of the body, I think he would have found it much easier to teach his methods; at all events his teachings would have taken firmer hold in less time. Being perfectly familiar with the criticism and comments I tried in various ways to make the inside way clear to my assistants and pupils. Holding a knee and assisting was the best thing I could do for my pupils. The modern crutch or knee holders were inadequate, so my plan is to have an assistant support and hold each knee firmly.

Emmet's inside operations are always pleasing, resulting in a strong resistant perineum, absence of rectocele, posterior vaginal wall hugging the anterior well up and a total absence of previous symptoms, such as fulness, weight, and pressure and a sensation of something protruding. The numerous failures in attempts at closure of vesicovaginal fistula and sphincter rents, freely demonstrated the loss of interest and the importance of apprenticeship or preparation to deal with such injuries successfully. In nearly all of the sphincter lesions and vesicovaginal fistulæ I deal with, one or more attempts have been made at closure. In some cases I find a good strong perineal band beneath a good sized fistula and, of course, a great disappointment at the result. Such failures strongly impress me with the importance of prolonged apprenticeship in this special line of surgical work in all educational centers, and, besides, the specialty should be encouraged in all well regulated and well managed hospitals.

Just at this point I want to pay my respects to the general surgeon. The general surgeon knows as much about gynecology as a Chinaman does about teaching Sunday school and he wholly forgets that his mother, his wife, his sister and his daughter are going to be neglected, and are going to live the lives of invalids incapacitated by easily correctible troubles, because he is an obstructionist, opposing this and other important specialties in the larger church and general hospitals. Again, he forgets the importance of his work while, metaphorically speaking, he does not stick to his last. If I were a young man I would go back to general surgery. I realise that general surgeons are greatly needed all over the country. In every town and village in which I enter I am asked to do some important general surgical operation, because it has been neglected; the patient has been passed by, turned down, or a shirking effort made. In one case I was asked to remove the prostate after two efforts had been made,—one above and one below. I found the enucleation as easy as rolling off a log, but the lower route of this operation which the general surgeon usually takes is fearful.

Let us encourage the specialist in all educational centers. The best is bad enough in every specialty. When we have vision corrected we seek the best ophthalmologist; famous artists rarely have more than one pupil. The operator should do plastic surgery early in the day when he is perfectly fresh and has ample time to do it well. He commonly does abdominal work and ends his clinic hurriedly with plastic surgery. He should have his plastic days and his pelvic and abdominal days, and do his work more carefully and better. After dabbling with dirty pelvic suppurative work, and extirpations for malignancy, he should not attempt surgery in healthy lymph spaces. Plastic surgery is too important to be done in a perfunctory manner; it encourages the spectator in shirking object lessons. I met two visitors on the street one day and asked them to come in the following morning to witness some plastic surgery. One replied: "Oh, h—l; I don't want to see that. Have you no celiotomies?" I relate this truthful anecdote to show you how little interest the visiting practitioner manifests in this important work. How greatly must his community be neglected!

241 N. EIGHTEENTH ST.

